# Binge eating disorder, emotional eating and night eating syndrome: A comparative study between subjects with normal weight, overweight and obesity

**DOI:** 10.1192/j.eurpsy.2021.959

**Published:** 2021-08-13

**Authors:** G. Esgalhado, S. Marques, H. Pereira

**Affiliations:** Psychology And Education, University of Beira Interior, Covilha, Portugal

**Keywords:** Night Eating Syndrome, emotional eating, binge eating disorder

## Abstract

**Introduction:**

Obesity has been associated with certain psychiatric disorders, especially in patients seeking treatment. It is known that obesity is not a psychiatric disorder in itself, however, it should be noted that a significant part of the population has some type of clinical eating disorder.

**Objectives:**

This study aims to assess levels of Binge Eating Disorder, Night Eating Syndrome and Emotional Eating patterns, according to different groups of Body Mass Index categories.

**Methods:**

A sample of 220 subjects, aged between 18 and 81 years old, with an average age of 33 years participated in this study. 140 (63.6%) were female and 80 were male (36.4%) The sample was divided into three comparison groups, according to the Body Mass Index (BMI). The following measures were used: Sociodemographic questionnaire, Binge Eating Scale, Night Eating Habits Questionnaire, and the Emotional Eating Scale.

**Results:**

When comparing the different BMI groups, it was found that obese subjects were the ones that most reported binge eating behavior. Overweight subjects had higher levels of binge eating when compared to normal-weight participants, but this was not true for Night Eating habits. Subjects with normal weight reported more nocturnal eating behavior, followed by obese individuals.

**Conclusions:**

The prevalence of binge eating disorder seems to be more frequent in obese people, tending to increase according to the level of severity of obesity. Normal-weight subjects reported more nocturnal eating behavior, so they may be more likely to develop this syndrome and, consequently, overweight or obesity.

